# Evaluation of Turmeric Nanoparticles as Anti-Gout Agent: Modernization of a Traditional Drug

**DOI:** 10.3390/medicina55010010

**Published:** 2019-01-11

**Authors:** Mubin Mustafa Kiyani, Muhammad Farhan Sohail, Gul Shahnaz, Hamza Rehman, Muhammad Furqan Akhtar, Irum Nawaz, Tariq Mahmood, Mobina Manzoor, Syed Ali Imran Bokhari

**Affiliations:** 1 Department of Bioinformatics and Biotechnology, Faculty of Basic and Applied sciences, International Islamic University Islamabad, Islamabad 44000, Pakistan; mubin3us@yahoo.com (M.M.K.); hamzarehman51@gmail.com (H.R.); 2 Riphah Institute of Pharmaceutical Sciences, Riphah International University, Lahore 54000, Pakistan; mfurqan.akhtar@riphah.edu.pk; 3 Department of Pharmacy, Faculty of Biological Sciences, Quaid-i-Azam University, Islamabad 44000, Pakistan; gshahnaz@qau.edu.pk; 4 Faculty of Rehabilitation and Allied Health Sciences, Riphah International University, Islamabad 44000, Pakistan; irum.nawaz@riphah.edu.pk; 5 Department of Nanoscience and Technology, National Centre for Physics, Islamabad 44000, Pakistan; tariqm20002000@yahoo.com; 6 Department of Pharmacy, Lahore College for Women University (LCWU), Lahore 54000, Pakistan; mobina_star@hotmail.com

**Keywords:** turmeric, nanoparticles, gout, monosodium urate crystals

## Abstract

*Background and objectives*: Turmeric has assisted in the control of inflammation and pain for decades and has been used in combination with other nutraceuticals to treat acute and chronic osteoarthritis pain. Recently, the effect of turmeric, turmeric extract, or curcuminoids on musculoskeletal pain, either by themselves or in conjunction with other substances, has been reported. The aim of this study was to develop and characterize turmeric nanoparticles (T-NPs) for various parameters, both in vitro and in vivo. *Materials and Methods*: The T-NPs were successfully synthesized and characterized using particle size analysis, solubility improvement, SEM, EDX, X-ray diffraction, and in vivo antigout activity in mice model. *Results*: The T-NPs were of about 46 nm in size with a positive zeta potential +29.55 ± 3.44 and low polydispersity index (PDI) (0.264). Furthermore, the diseased mice, with induced gout via monosodium urate crystals, were treated with 5, 10, and 20 ppm T-NPs, administered orally, and the anti-gout potential was observed through measurement of joint diameter and changes in biochemical parameters, including lipid profile, renal function test, and liver function tests which significantly reduced the levels of these biochemical parameters. *Conclusions*: Uric acid levels were significantly reduced after the treatment with T-NPs. indicating that T-NPs show superior potential against gout management. Thus, T-NPs can be developed as an efficient antigout agent with minimum toxicities.

## 1. Introduction

Gout, a metabolic disorder, develops due to the excessive production of uric acid in the body as a result of purine metabolism which gets deposited into the joints as monosodium urate crystals. Most frequently, gout causes recurrent attacks of acute arthritis and may sometimes lead to chronic arthropathy, tophi depositions, and renal diseases [1,2]. The initial symptoms of gout usually consist of intense episodes of painful swelling in a single joint, most often in the feet, especially the big toe. It can affect the insteps, ankles, heels, knee, wrists, fingers, elbows, and other areas, causing pain, swelling, redness, heat, and stiffness in joints [3].

The current therapy for gout is to use either uricosuric agents or xanthine oxidase inhibitors (XOI), in order to block the synthesis of uric acid from purines; however, in an attempt to prevent the side effects of drugs, there is a need to develop natural compounds with XOI activity. Allopurinol is the most commonly prescribed XOI in the treatment of gout [4]. Conversely, herbal medicines are now extensively being studied for their therapeutic activity, due to the anti-inflammatory properties for arthritic patients [5]. Contemporary research on herbal medicine for arthritis focuses on the improvement in bioavailability, stability, and reduction in side effects of these herbal medicines, thus providing effective alternative medication for arthritis. Evidence suggests that the effectiveness of herbal actives can be improved by understanding the exact mechanism of action and by administering them through novel technologies, like liposomes, nanoparticles, phytosomes, and transdermal drug delivery [6].

Turmeric medicinally assists in the control of inflammation and pain, and contains at least three naturally occurring polyphenols, namely curcumin, demethoxycurcumin, and bisdemethoxycurcumin, collectively termed as curcuminoids. Polyphenols have been shown to have an antioxidant effect in tissues, and there appears to be an inverse relationship between the consumption of a polyphenol-rich diet and the occurrence of chronic disease. Various studies describe the total curcuminoids by percentage in the turmeric root as ranging between 3% and 6% of dry weight. Curcumin is the most prevalent curcuminoid found in turmeric, making up around 77% of the total curcuminoids in the plant. Desmethoxycurcumin makes up about 17% of the total curcuminoids, and bisdemethoxycurcumin about 5% [7].

Herbal therapies have been in practice since ancient times, and their effectiveness lies in the supply of active ingredient. Most of the active ingredients from plants like flavonoids, alkaloids, and terpenoids are hydrophilic in nature, but less permeable to membrane barriers, declared as a rate-limiting step for these drugs [8]. Nanotechnology has opened new avenues to developing formulations with improved pharmacokinetics, pharmacodynamics, and therapeutic outcomes. Several nano-based formulations (liposomes, polymeric nanoparticles, solid lipid nanoparticles (SLNPs), nanoemulsions, and more) containing traditional herbs have been reported with tremendously improved properties and diverse application with targeted delivery [9,10,11].

The current project aimed to examine the effect of turmeric nanoparticles as an agent for therapy against gout. The nanoparticles were successfully developed and characterized physiochemically to observe their effect on pharmacological activities. A gout model was developed in mice, and the efficacy of turmeric nanoparticles at varying concentrations was evaluated in vivo, in comparison with standard gout therapy.

## 2. Results and Discussion

### 2.1. Characterization of MSU Crystals

The monosodium urate (MSU) crystals were successfully developed via the utilized method. The prepared crystals appeared as characteristic needle-shaped negative birefringence under a light polarized microscope. The size appeared to be around 30 µm [12]. The developed MSU crystals were injected to introduce gout into the mouse model.

### 2.2. Characterization of Turmeric Nanoparticles

**Figure 1:**
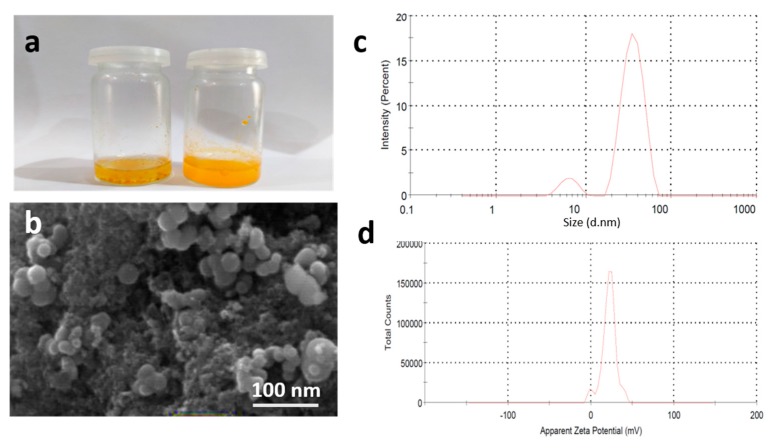
(**a**) Solubility of turmeric powder (left vial) and turmeric nanoparticles (T-NPs) (right vial). (**b**) Scanning electron microscope image of synthesized turmeric NPs showing smooth spherical nanoparticles with uniformity in size distribution, and (**c**) size distribution and (**d**) zeta potential distribution of T-NPs.

**Figure 2:**
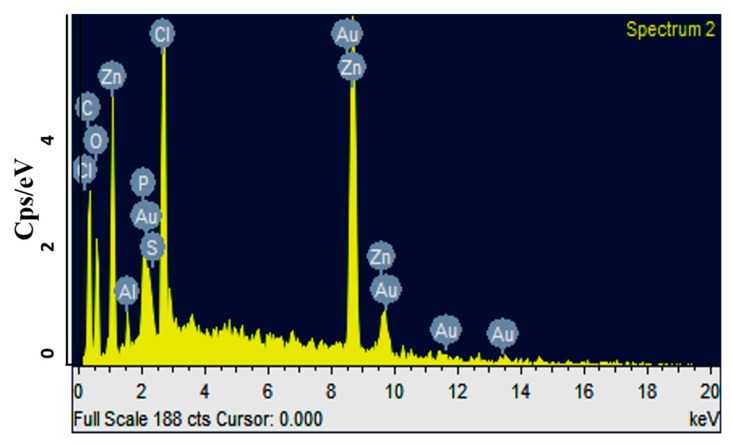
Energy-dispersive X-ray spectroscopy (EDX) spectrum of turmeric nanoparticles (T-NPs), showing ratios of different metals present in the sample.

T-NPs were successfully prepared using a previously described synthesis protocol [13]. The hydrodynamic particle size was observed to be 46 ± 2.43 nm using dynamic light scattering (DLS) technique. The zeta potential plays an important role in understanding the state of particle surface properties. Not only does it determine the stability of nanoparticles, but it also defines the possible interaction of nanoparticles with various plasma membranes. The results showed that particles had a zeta potential of +29.55 ± 3.44, suggesting that their higher stability and interaction with anionic plasma membranes was owing to the + charge. Moreover, relatively low values of polydispersity index, i.e., 0.264, indicated uniformity in the synthesis of nanoparticles. The T-NPs were aimed to achieve improved solubility following oral administration. The solubility was assessed by preparing 2 mL (1 mg/mL) solution of both the turmeric powder and T-NPs. The turmeric powder showed partial solubility with solid residue at bottom of the vial, and T-NPs showed no residue at the bottom, as shown in Figure 1a. The particles appeared spherical in shape, with a smooth surface upon characterization using SEM, as shown in Figure 1. Moreover, energy-dispersive X-ray spectroscopy (EDS) of T-NPs was performed (Figure 2), which revealed the amounts of different elements that constitute turmeric.

### 2.3. XRD Analysis

**Figure 3:**
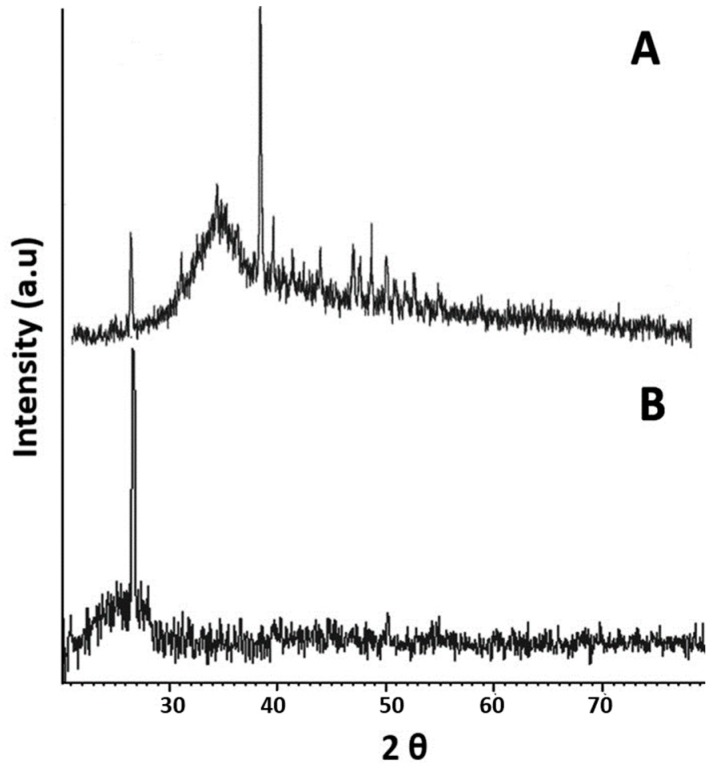
(**A**) XRD (X-ray diffraction) spectra of turmeric powder showing peaks and amorphous nature of turmeric, (**B**) XRD analysis of synthesized turmeric nanoparticles (T-NPs) showing a prominent sharp peak at 26.7176° of 2*θ* corresponding to the intensity of 327, indicating the presences of turmeric particles.

The XRD analysis was performed to study the nature of prepared T-NPs. The XRD spectra of T-NPs was compared to turmeric powder (Figure 3A), which showed characteristic peaks between 2*θ* 30–40 mainly for curcumin and other ingredients in crystalline form. However, the peaks disappeared in T-NPs, indicating the presence of turmeric and its constituents in amorphous nature. This amorphous nature of T-NPs resulted in improved solubility. Also, a slight shift in 2*θ* peaks of T-NPs was observed, which might indicate the conversion of turmeric/curcumin into nanoparticles, which slightly changed the amorphous nature of turmeric powder [14]. The conversion to amorphous nature resulted in diminished peaks. The results (Figure 3B) showed the amorphous nature of T-NPs with a single characteristic peak at 26.7176° of 2*θ* at the x-axis, corresponding to the intensity of 327 nm, indicating the presences of turmeric particles [11]. The Debye-Scherrer equation was utilized to calculate the size of the formulated T-NPs, which was around 36.12 nm, corresponding to the size calculated through SEM (46 nm).

### 2.4. Evaluation of Anti-Gout Potential

#### 2.4.1. Induction of Gouty Arthritis in Mice

Induction of gouty arthritis in mice was successfully achieved by intraarticular injection of MSU crystals into the left ankle of mice. After 3 weeks of successful induction, the swelling of mice left ankles was confirmed by measuring using a Vernier caliper (Figure 3), which confirmed a significant increase in the swelling of ankles, thus indicating gouty arthritis.

#### 2.4.2. In Vivo Studies

Turmeric has demonstrated its medicinal importance for centuries, and has been used in the ayurvedic treatment of several ailments, including diarrhea, stomach ulcer, skin inflammation and aging, diabetes, neurodegenerative disorders, and arthritis, acting as an anti-inflammatory agent. The major constituent of turmeric that is of medicinal importance is curcumin, having anti-inflammatory and antineoplastic activity [15]. Turmeric acts mainly by modifying NF-κB signaling and proinflammatory cytokine (IL, phospholipase A2, 5-LOX) activity, thus reducing joint inflammation [16]. The water-insolubility of turmeric powder is a rate-limiting step for oral absorption, thus, limits its oral administration. T-NPs were aimed to overcome this barrier and increase oral bioavailability by crossing enterocytes and reaching systemic circulation to impart its anti-gout activity. To observe the improvement in the anti-inflammatory response of T-NPs in MSU-induced mice, T-NPs were orally given in various concentrations. The diseased mice were subjected to the treatment with newly developed T-NPs at varying concentrations, i.e., 5, 10, and 20 ppm. The treatment was compared with a standard anti-gout agent, allopurinol, at a dose of 100 mg/kg. The treatment was continued for 3 weeks, and during that, mice were observed for any changes in behavior, health, body weight, and other parameters, which showed no evidence of changes during the whole course of treatment. After 3 weeks, biochemical analyses on the blood of mice were performed to see the effect of all treatment protocols, and were compared with the control group.

Liver function tests (Table 1) showed that alanine aminotransferase (ALT) and aspartate aminotransferase (AST) values for the control group were within the normal range, which increased significantly in the diseased group (*p* < 0.05). The treatment with allopurinol and T-NPs (5 ppm) decreased the values close to the upper border limit of normal range (40 U/L). Total bilirubin showed no significant deviation in any group as compared with control, showing all values within the acceptable ranges. Alkaline phosphatase (ALP) showed significant variation in all groups, though it remained within the accepted limit. Treatment with T-NPs at the lowest dose of 5 ppm dramatically decreased ALP level, as compared with higher treatment groups (10 and 20 ppm), which did not decrease significantly. This decrease could account for the malfunctioning of liver or any obstruction in bile duct, leading to the decreased release of ALP in blood. Previous studies have shown that MSU induces oxidative stress, resulting in liver damage, as evidenced by a rise in ALT and AST [17]. However, treatment with T-NPs exhibited a significant decrease in both parameters, especially at 20 ppm. The hepatoprotective effect of turmeric has been previously documented and may be attributed to the presence of phenolics, flavonoids, and curcumin.

Evaluation of lipid profile (Table 2) revealed that T-NPs significantly (*p* < 0.05) lowered the cholesterol, triglycerides, and low-density lipoprotein (LDL) levels in arthritic mice at 10 and 20 ppm. Treatment with T-NPs at 10 and 20 ppm also brought the level of cholesterol and LDL within normal, in contrast to allopurinol therapy, which failed to reduce elevated levels of cholesterol, LDL, and triglycerides. The lipid-lowering effect of turmeric is mainly due to the presence of curcumin. The cholesterol-lowering effect of curcumin has been well documented previously, acting to reduce hyperlipidemia mainly through ameliorating oxidative stress and modulating signal transduction and transcription factors [18]. Furthermore, this T-NP-induced decline in cholesterol and LDL may be helpful in preventing diabetes in arthritic patients via reducing insulin resistance and modulating inflammatory cytokines [19].

Renal function tests (RFTs) included evaluation of urea, uric acid, and creatinine. The results (Table 3) indicated that urea and creatinine increased in the diseased group compared to control, yet remained within normal values. All treatments decreased the level of urea and uric acid as compared to diseased control group, indicating the success of the therapy. The results for uric acid indicated that all the treatment groups showed a highly significant lowering of uric acid levels in the blood, which indicated suppression of hyperuricemia and, thus, treatment of gout. The treatment with T-NPs was most efficient among all treatment groups as it decreased the uric acid level significantly lower (1.5 ± 0.41 mg/dL) even lower than normal values (4–10 mg/dL). This decrease was even better than a reported study where curcumin reduced the uric acid level to 2.93 ± 0.38 mg/dL, significantly (*p* ≤0.001) [4].

The complete blood picture (Table 4) was evaluated to see the effect of treatment on blood components. For the diseased group, all parameters were significantly higher as compared to the control group. White blood cells increased to 34.33 ± 4.86 × 10^3^/mm^3^ which, upon treatment with T-NPs, decreased to normal values (6–7 × 10^3^/mm^3^).

The results indicated a significant decrease (*p* ≤0.05) in the uric acid level of all treated groups 1.5 ± 0.41, 2.16 ± 0.47, and 2.0 ± 0.81 with T-NPs at 5, 10, and 20 ppm respectively showing a tremendous ability to decrease serum uric acid levels. This decrease was much better than what has been reported with coumarin (0.5 mg/kg) and allopurinol (10 mg/kg) in mice having hyperuricemia, where serum uric acid levels decreased to 3 ± 0.26 mg/dL (*p* ≤0.001) and 5.37 ± 0.27 mg/dL (*p* ≤0.001) respectively, in comparison to control group [2].

#### 2.4.3. Histopathological Evaluation

**Figure 4:**
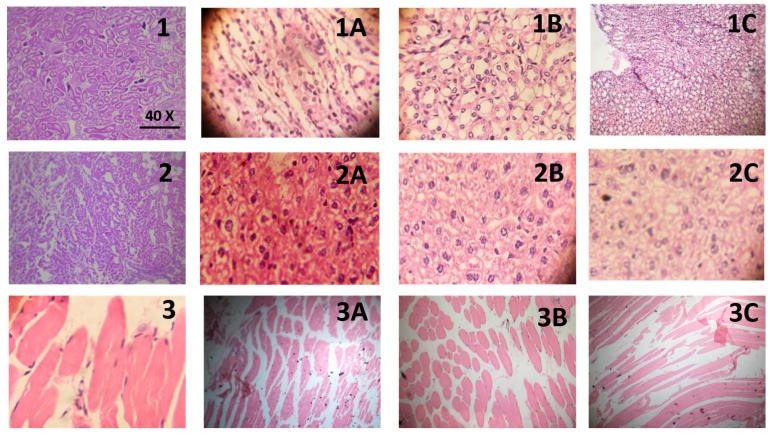
Histopathological slides of all treatment groups in which Series 1 represents kidney, where 1 is the control group, (**1A**) treatment with 5 ppm T-NPs, (**1B**) treatment with 10 ppm T-NPs, (**1C**) treatment with 20 ppm T-NPs; Series 2 represents liver, where 2 is the control group, (**2A**) treatment with 5 ppm T-NPs, (**2B**) treatment with 10 ppm T-NPs, (**2C**) treatment with 20 ppm T-NPs; and Series 3 represents muscle, where 3 is the control group, (**3A**) treatment with 5 ppm T-NPs, (**3B**) treatment with 10 ppm T-NPs, (**3C**) treatment with 20 ppm T-NPs. All images were taken at 40× optical zoom.

To further probe the effects of T-NPs, histopathology of the liver, kidney, and muscles was performed. Photomicrographs of kidney sections revealed that there was some evidence of a decrease in epithelial cells of proximal convoluted tubules in untreated disease control mice. However, treatment with T-NPs at 5, 10, and 20 ppm, or allopurinol, reduced the damage to the renal epithelial cells (Figure 4). There was no deteriorative effect on the muscle histology of the untreated disease group mice as compared with the control group. Treatment with T-NPs also did not exhibit any alteration in histology of muscle fibers.

There was a mild degree of necrosis in hepatocytes of mice exposed to MSU as compared with the normal control group. Treatment with the T-NPs at 5, 10, and 20 ppm, or allopurinol, reduced the MSU-induced necrosis in hepatocytes as shown in Figure 4. The exact mechanism of MSU-induced nephrotoxicity and hepatotoxicity is unknown. However, the MSU-induced damage to the kidney epithelial cells and hepatocytes is mainly attributed to the formation of the uric acid crystals and inflammasomes that damage the membranes of lysosomes, culminating in the release of inflammatory mediators and oxidative stress [20]. It has previously been shown that the polyphenols and curcumin present in turmeric powder prevent the release of inflammatory cytokines such as IL-1β, and formation of inflammasomes responsible for mitochondrial dysfunction [21]. Therefore, the protective effect of T-NPs against MSU-induced nephrotoxicity and hepatic necrosis may be due to curcumin and polyphenolic compounds, such as catechin present in turmeric [22].

## 3. Material and Methods

### 3.1. Materials

Turmeric powder was purchased from the local market. All the solvents and chemicals used were of analytical grade and were purchased from Sigma-Aldrich, Darmstadt, Germany.

### 3.2. Methods

#### 3.2.1. Preparation of MSU Crystals

Synthesis of monosodium urate (MSU) crystals was done following a reported method with slight modifications. Briefly, 4 g of uric acid was dissolved and heated in 800 mL of H_2_O with NaOH (9 mL, 0.5 N), and the pH was adjusted to 8.9 at 60 °C. The mixture was cooled overnight in a cold room, then washed and dried. Needle-like crystals were recovered and were suspended in sterile saline at 20 mg/mL [23].

#### 3.2.2. Synthesis of Turmeric Nanoparticles

Turmeric nanoparticles (T-NPs) were synthesized following a reported method with slight modifications [13]. Briefly, 5 mg of turmeric powder was dissolved in 20 mL of dichloromethane. From this stock solution, 1 mL was added dropwise to 50 mL boiling water under ultrasonification with a power and frequency of 20 kHz using bath sonicator (SJIA-W-2, Zhejiang, China) for 60 min. After this, the solution was mixed at 800 rpm for 20 min. The sample was centrifuged, and the pellet of T-NPs was collected. The obtained pellet was dried for further use. The solubility of the T-NPs was assessed by dissolving the weighed quantity of T-NPs in water at 37 °C ± 1.0, and compared with same amount of turmeric powder.

#### 3.2.3. Particle Size, PDI, and Zeta Potential of NPs

The hydrodynamic size, surface zeta potential, and polydispersity index (PDI) of T-NPs were evaluated using a Zetasizer Nano (Malvern, Malvern, UK) [24]. The sample was diluted 10-fold before taking readings through the Zetasizer. The cuvette was filled with 1 mL of T-NP sample. The particle size was measured at 25 °C using He–Ne laser as a light source and avalanche photodiode (APD) as a detector. For zeta potential measurement, the electrophoretic light scattering technique was used. The results were interpreted using Malvern Zetasizer software [25,26].

#### 3.2.4. Scanning Electron Microscopy and EDX Analysis of NPs

The surface morphology and particle size of T-NPs were evaluated using a scanning electron microscope (TESCAN VEGA 3, Pennsylvania, USA). The samples were carefully prepared by applying a diluted sample on a glass slide and dried, followed by gold coating using a sputter coater (Denton, Desk V HP, Farmers Branch, TX, USA) operating at 40 mA for 20 s under vacuum. The samples were analyzed at 20 kV, focusing at 500 nm scale. Moreover, energy-dispersive X-ray (EDX) was performed to quantify the elements in T-NPs [27].

#### 3.2.5. XRD Analysis of NPs

The structural nature of the T-NPs was evaluated using XRD (Perkin-Elmer FTIR-1600, Massachusetts, USA). The samples were run between 20 and 70 2*θ* [28]. The particle size of T-NPs was also calculated, using the Debye-Scherrer equation given below.

(1) $$\text{Debye-Scherrer equation}=\tau =\frac{K\lambda }{\beta \;\cos\theta }$$

#### 3.2.6. Evaluation of Anti-Gout Potential

##### Induction of Gouty Arthritis in Mice

Induction of gouty arthritis in mice was achieved by injecting developed MSU crystals (described earlier) intraarticularly in the left ankle of mice, at one-day intervals, for 3 weeks. Intraarticular-injected MSU was discontinued and followed by intraperitoneal injection of MSU for 2 weeks to induce gout in mice.

##### In Vivo Studies

The animal studies were conducted as per approved guidelines from the research ethical committee of International Islamic University, Islamabad, Pakistan (Letter No. IIU(BI&BT)/FBAS-IBBC-2015-4). BALB/c mice, 25–30 g, of either sex, were obtained from National Institute of Health (NIH) Islamabad, Pakistan and were acclimatized for a week in a light- and temperature-controlled room with a 12 h dark–light cycle, and fed with commercial pelleted feed from NIH, and water was freely available. The mice were randomly divided into six groups (n = 6). Group I served as a control group (without disease). Group II (with the disease) served as a positive control. Group III (with the disease) was treated with allopurinol (100 mg/kg), and group IV, V, and VI (all with the disease) were respectively treated, orally, with 5, 10, and 20 ppm T-NPs.

##### Biochemical Analysis

After treatment with various formulations for 3 weeks, the gouty mice animals were subjected to biochemical and histopathological evaluations. The blood was collected from mice via cardiac puncture and collected in two different vials for the evaluation of various blood parameters, including LFTs, RFTs, complete blood picture CBP, and lipid profiles. [29].

##### Histopathological Evaluation

After blood collection, the mice were euthanized and the liver, kidney, and muscles were carefully removed. The organs and tissues were washed with normal saline and fixed in a paraffin block. The tissue sections (3–5 µm) were prepared using microtome, and microscopically evaluated [30].

#### 3.2.7. Statistical Analysis

The results obtained from biochemical tests were compared by SPSS (version 25). One-way ANOVA and Bonferroni’s post-hoc test were used for the analyses of the experimental data, where data were considered statically significant at *p* < 0.005.

## 4. Conclusions

The T-NPs were successfully developed and characterized as an anti-gout agent with significant uric acid-lowering ability. The gouty mice treated with orally administered T-NPs at various concentrations showed a significant concentration-dependent reduction in uric acid levels, along with a reduction in the lipid profiles of the mice. Furthermore, complete blood picture and histopathology of organs revealed the safety of the formulations following 3 weeks of treatment with T-NPs. Hence, the study concluded that T-NPs can overcome the intestinal barriers of orally administered turmeric powder, and can serve as a promising anti-gout agent as compared to turmeric powder and allopurinol therapy, with minimum toxicities and improved patient compliance.

## Figures and Tables

**Table 1:** Liver function tests (LFTs) performed on the plasma of various groups after three weeks of treatment with turmeric NPs (T-NPs) in comparison with standard allopurinol (Allp) treatment. The results are shown as mean ± SD of 6 experiments, and *p* < 0.05 were considered statistically significant.

Parameter	Unit	Normal Range	Control Group	Diseased Group	Allp-Treated Group (50 mg)	T-NPs Treated Group (5 ppm)	T-NPs Treated Group (10 ppm)	T-NPs Treated Group (20 ppm)
ALT	U/L	10–40	34 ± 6.48 ^a^	61.16 ± 9.40 ^b^	51 ± 5.01 ^b^	48 ± 9.16	63 ± 5.35 ^b^	42 ± 11.3
AST	U/L	10–40	36 ± 3.55 ^a^	55.83 ± 7.31 ^b^	45.16 ± 2.13	49 ± 5.53	68 ± 2.58 ^b^	50 ± 3.21 ^b^
Total bilirubin	mg/dL	0.2–1.0	0.46 ± 0.16	0.41 ± 0.10	0.41 ± 0.09	0.5 ± 0.16	0.5 ± 0.12	0.6 ± 0.08
ALP	U/L	65–306	189 ± 22.99	183.33 ± 53.60	171 ± 18.97	69 ± 3.78 ^ab^	328 ± 35.59 ^ab^	142 ± 31.28

Where “a” and “b” showed a statistically significant difference when the treatments were compared with the disease and control groups, respectively. ALT-alanine aminotransferase. AST-aspartate aminotransferase. ALP-alkaline phosphatase.

**Table 2:** Lipid profile evaluation of various groups after three weeks of treatment with turmeric NPs (T-NPs) in comparison with standard allopurinol (Allp) treatment. The results are shown as mean ± SD of 6 experiments, and *p* < 0.05 were considered statistically significant.

Parameter	Unit	Normal Range	Control Group	Diseased Group	Allp-Treated Group (50 mg)	T-NPs Treated Group (5 ppm)	T-NPs Treated Group (10 ppm)	T-NPs Treated Group (20 ppm)
Cholesterol	mg/dL	<200	146.66 ± 35.51	207.33 ± 32.92	135.5 ± 29.78	142 ± 23.49	87 ± 13.06 ^a^	85 ± 4.51 ^ab^
HDL	mg/dL	35–65	34.66 ± 14.42	24.33 ± 2.74	23 ± 0.89	28 ±4.32	20 ± 2.58	20 ± 1.63
LDL	mg/dL	<150	68 ± 8.6	108.16 ± 50.32	61 ± 11.67	62 ± 12.94	14 ± 1.29 ^ab^	15.16 ± 1.95 ^ab^
Triglycerides	mg/dL	<150	122 ± 22.75 ^a^	298.66 ± 47.43 ^b^	306 ± 61.69 ^a^	331.66 ± 44.13 ^a^	215 ± 38.11 ^a^	331 ± 12.93 ^a^

Where “a” and “b” showed a statistically significant difference when the treatments were compared with the disease and control groups, respectively. HDL-high-density lipoprotein. LDL-low-density lipoprotein.

**Table 3:** Renal function tests (RFTs) performed on the plasma of various groups after three weeks of treatment with turmeric NPs (T-NPs) in comparison with standard allopurinol (Allp) treatment. The results are shown as mean ± SD of 6 experiments, and *p* < 0.05 were considered statistically significant.

Parameter	Unit	Normal Range	Control Group	Diseased Group	Allp-Treated Group (50 mg)	T-NPs Treated Group (5 ppm)	T-NPs Treated Group (10 ppm)	T-NPs Treated Group (20 ppm)
Urea	mg/dL	10–50	33 ± 7.25^a^	61.5 ± 7.04 ^b^	38.16 ± 3.60 ^a^	14.5 ± 2.21 ^ab^	35 ± 5.94 ^a^	10 ± 2.51 ^ab^
Creatinine	mg/dL	0.5–1.3	0.63 ± 0.12	0.98 ± 0.22	0.41 ± 0.14 ^a^	0.4 ± 0.21 ^a^	0.32 ± 0.26 ^a^	0.3 ± 0.15 ^a^
Uric Acid	mg/dL	4–7	4.63 ± 0.69 ^a^	9.8 ± 1.29	2.11 ± 0.37 ^ab^	1.5 ± 0.41 ^ab^	2.16 ± 0.47 ^ab^	1.55 ± 0.13 ^ab^

Where “a” and “b” showed a statistically significant difference when the treatments were compared with the disease and control groups, respectively.

**Table 4:** Complete blood picture of animal groups after three weeks of treatment with turmeric NPs (T-NPs) in comparison with standard allopurinol (Allp) treatment. The results are shown as mean ± SD of 6 experiments, and *p* < 0.05 were considered statistically significant.

Parameter	Unit	Normal Range	Control Group	Diseased Group	Allp-Treated Group (50 mg)	T-NPs Treated Group (5 ppm)	T-NPs Treated Group (10 ppm)	T-NPs Treated Group (20 ppm)
WBCs Count	10^3^/mm^3^	4–10	5.33 ± 0.91 ^a^	34.33 ± 4.86 ^b^	3.66 ± 0.56 ^a^	6.0 ± 0.74 ^a^	7.0 ± 0.86 ^a^	6.6 ± 0.61 ^a^
RBCs Count	mL/mm^3^	4.50–6.00	6.193 ± 0.22	7.12 ± 1.20	6.34 ± 0.02	8.74 ± 0.17	7.795 ± 0.31	7.82 ± 0.18
Hemoglobin	g/dL	11.0–15.0	11.5 ± 0.96	8.83 ± 1.99	8.76 ± 2.12	14.78 ± 0.42 ^ab^	14.28 ± 0.46 ^ab^	12.4 ± 0.62
Hematocrit	%	40–50	36 ± 4.32	34 ± 8.46	30.83 ± 5.67	47.16 ± 4.05	46 ± 1.29	44 ± 4.54
MCV	Fl	76–92	80 ± 2.43 ^a^	47.83 ± 4.66 ^b^	47.66 ± 3.50 ^b^	54 ± 5.85 ^b^	59 ± 1.82 ^b^	56 ± 5.62 ^b^
MCH	Pg	23–31	27.33 ± 2.49 ^a^	14.83 ± 2.03 ^b^	14.16 ± 2.04 ^b^	17 ± 1.41 ^b^	18 ± 1.0 ^b^	16 ± 2.08 ^b^
MCHC	g/dL	32–36	30.66 ± 1.24	32.66 ± 5.05	31 ± 2.75	31 ± 2.38	31 ± 0.81	28 ± 2.31
RDW CV	%	11.5–16.0	18.66 ± 3.29	21.83 ± 2.79	21 ± 3.84	16 ± 1.29	19 ± 4.47	17 ± 1.00
Platelet Count	10^3^/mm^3^	150–450	231.7 ± 77.15	182.7 ± 42.71	1040 ± 44.3 ^ab^	554± 247.3	240 ± 37.02	771.66 ± 80.06 ^ab^

Where “a” and “b” showed a statistically significant difference when the treatments were compared with the disease and control groups, respectively.
